# From benchmark accuracy to pharmacological credibility: why mechanistic explainability must define the next generation of AI for drug repurposing

**DOI:** 10.3389/fphar.2026.1860953

**Published:** 2026-07-30

**Authors:** Markus Bertl, Salvatore Sinno

**Affiliations:** 1 Advanced Research and Innovation Group, Unisys, Blue Bell, PA, United States; 2 Department of Information Systems and Operations Management, Vienna University of Economics and Business, Vienna, Austria; 3 School of Computing, Newcastle University, Newcastle upon Tyne, United Kingdom

**Keywords:** biomedical knowledge graphs, drug repurposing, explainable artificial intelligence (XAI), graph neural networks (GNNs), mechanistic interpretability, translational pharmacology, uncertainty quantification

## Abstract

Artificial intelligence (AI) is increasingly used in drug repurposing to integrate chemical, biological, and clinical data and to prioritize candidate drug–disease associations. Yet current evaluation practices remain dominated by benchmark metrics such as AUROC, AUPRC, F1, or top- k retrieval, which do not by themselves establish pharmacological credibility. A useful prediction is not merely one that scores highly on retrospective datasets, but one that can be connected to a biologically plausible mechanism, safety-relevant context, and a traceable evidentiary basis for experimental follow-up. Mechanistic explainability should therefore be treated as a core objective for translationally oriented AI-enabled repurposing, while also emphasizing that explainability complements rather than replaces experimental validation. Specifically, explanations should connect drugs, targets, pathways, phenotypes, and clinical outcomes in ways that are intelligible to pharmacologists and compatible with experimental prioritization, translational decision-making, and emerging regulatory expectations. We identify a central gap in the literature: many explainability methods emphasize feature attribution or local model transparency, but rarely produce pharmacology-aligned evidence chains that also communicate uncertainty, robustness, and provenance. We introduce biomedical knowledge graphs and neurosymbolic approaches as a promising foundation for more credible repurposing systems because they can support relational inference, structured mechanistic reasoning, and provenance-aware explanation. We further argue that future evaluation should extend beyond predictive accuracy to include mechanistic coherence, uncertainty, robustness, and provenance (MURP) for experimental pharmacology, ideally within workflows that integrate computational prediction with laboratory and, where feasible, real-world validation. Reframing success in this way would improve rigor, translational relevance, and regulatory readiness in AI-driven drug repurposing.

## 1 Introduction

Artificial intelligence (AI) has increasingly become a catalyst for accelerating drug repurposing by integrating vast chemical, biological, pharmacological, and clinical data. Models that leverage these heterogeneous sources can rapidly propose drug–disease hypotheses and identify mechanistic leads that traditionally require substantial time and human labor (Pushpakom et al., 2019; Zhang et al., 2025). Yet, despite this promise, the field remains dominated by an “accuracy-first” mindset in which performance metrics such as area under the receiver operating characteristic (AUROC), area under the precision–recall curve (AUPRC), or top- k retrieval accuracy are treated as primary indicators of scientific value. These metrics are useful for benchmarking algorithmic progress, but they do not equate to pharmacological credibility. A link predicted with high probability on a retrospective dataset is not necessarily biologically meaningful, clinically actionable, or safe to advance.

This misalignment has become increasingly salient as regulatory bodies sharpen their expectations for transparency and trustworthy AI. In January 2026, the European Medicines Agency (EMA) and United States Food and Drug Administration (FDA) introduced joint *Guiding Principles of Good AI Practice in Drug Development* ^1^, emphasizing transparency, risk-based lifecycle management, clear communication of model capabilities and limitations, and the need for traceable evidence to support AI-informed decision-making. These principles are not specific to drug repurposing; rather, they address AI across the broader medicines lifecycle. Nevertheless, they are directly relevant to repurposing contexts in which biological plausibility, safety, traceability, and human oversight are essential. The EMA–FDA principles explicitly call for clear and relevant information about model behavior, uncertainty, and limitations; repurposing predictions that lack mechanistic justification fail this requirement by design.

Mechanistic explainability in AI-enabled drug repurposing increasingly functions as a form of scientific and regulatory infrastructure rather than a purely interpretive aid. The EMA–FDA principles emphasize lifecycle governance, including transparency, traceability, change management, and post-deployment monitoring of model behavior. Under this framing, explainability is important not only for scientific understanding but also for enabling auditability and defensible decision-making as models evolve over time. For repurposing systems, this implies that predictions should be accompanied by mechanistic rationales that remain intelligible when models, data sources, or knowledge graph (KG) snapshots are updated. Structured, provenance-aware evidence chains directly support these expectations by making explicit the biological assumptions underlying a prediction. They also enable reviewers to assess how changes in data, model parameters, or graph structure affect mechanistic conclusions.

In practical drug repurposing workflows, these requirements translate into a need for predictions that clarify *why* a drug might modulate disease biology, through what mechanism, in which tissues or cell types, and with what anticipated liabilities. In the absence of this mechanistic visibility, even highly ranked suggestions pose challenges for experimental prioritization, trial design, and regulatory engagement.

We therefore propose a pharmacology-centered standard. First, current explainability approaches are often misaligned with the epistemic needs of pharmacology because they rarely produce biologically intelligible evidence chains. Second, KGs, graph-based learning, and neurosymbolic approaches provide a promising substrate for pharmacology-aligned explanation. Third, future evaluation should treat mechanistic coherence, uncertainty, robustness, provenance, and experimental actionability as central dimensions of model quality. We collectively refer to these dimensions as the MURP framework (mechanistic coherence, uncertainty, robustness, and provenance). As a Perspective article, this paper does not present a new benchmark, dataset, or executable pipeline, nor does it follow a systematic review methodology. Instead, it synthesizes representative methodological and translational examples from the literature to argue for a more rigorous standard of evaluation in AI-enabled drug repurposing.

## 2 Why benchmark accuracy is not enough for pharmacology

Although statistical performance metrics remain necessary, they are insufficient for judging whether an AI-generated repurposing hypothesis is scientifically viable. A top- k hit in a retrospective benchmark does not guarantee that the underlying biological rationale is sound. Many models learn shortcuts tied to network topology, database incompleteness, label leakage, or biased edge distributions (Kondratyeva et al., 2022; Singh et al., 2023; Brière et al., 2025). Without insight into the mechanistic chain linking a drug to a disease, researchers must rely on manual curation, which is slow, subjective, and prone to confirmation bias. Moreover, unexplained predictions impede translation: pharmacologists, clinicians, and regulatory reviewers require clarity on the molecular and physiological basis of proposed interventions.

Two factors amplify the need for mechanistic explainability. First, repurposing inherently draws on prior clinical knowledge about safety, off-target effects, pharmacokinetics, and contraindications. A candidate drug’s historical profile can either support or weaken confidence in a new indication, but only if the proposed rationale can be assessed against known biology. Second, clinical and translational decision-making is risk-sensitive. Understanding potential failure points or safety concerns early allows improved experimental design and more disciplined resource allocation. For these reasons, benchmark metrics should be viewed as one component of evaluation rather than a surrogate for pharmacological value.

It is important, however, to distinguish several related concepts that are often conflated in the literature. *Interpretability* typically refers to the extent to which a model’s internal workings are understandable to humans. *Explainability* refers more broadly to the extent to which a model’s outputs can be made understandable, whether intrinsically or by post-hoc methods. *Mechanistic plausibility* is a stronger requirement: it asks whether a computational explanation is consistent with known biological directionality, tissue or cell-type context, and pharmacological logic. *Experimental validation* is stronger still, requiring empirical confirmation through wet-lab studies, clinical data, or real-world evidence.

The central problem is therefore not simply that many current explainability methods are imperfect, but that they are often poorly matched to the epistemic needs of pharmacology. Generic post-hoc tools such as SHAP (Lundberg and Lee, 2017) or LIME (Ribeiro et al., 2016) may identify influential features, yet they rarely explain whether a predicted drug–disease association is consistent with known mechanism of action, pathway biology, tissue context, adverse-effect liabilities, or contraindication patterns. In drug repurposing, however, these are precisely the questions that determine whether a computational signal becomes a credible pharmacological hypothesis. A useful explanation should therefore resemble an evidence chain rather than a feature ranking: it should connect drug, target, pathway, phenotype, and clinical consequence in a traceable and biologically intelligible way, while also communicating uncertainty and provenance.

BOX 1 **Definition: pharmacological evidence chain**

We define a pharmacological evidence chain as an ordered, semantically constrained sequence of biomedical relations that provides a mechanistic rationale for a drug–disease association. In its canonical form, an evidence chain links:

drug→target→pathway→phenotype→clinical outcome.

Each edge in the chain may be annotated with uncertainty estimates and provenance metadata, and extended with safety-relevant relations such as adverse drug reactions, drug–drug interactions, or contraindications. Evidence chains thus encode not only connectivity, but structured, inspectable mechanistic reasoning suitable for pharmacological interpretation and experimental prioritization.

In practice, evidence chains can be represented as weighted paths or explanatory subgraphs, where each relation is associated with a confidence score reflecting uncertainty, robustness, or evidentiary strength. Canonical chains will often span four or five biologically meaningful hops, although shorter or longer chains may be acceptable when justified by the disease context. What matters is not path length alone, but whether the chain respects biological directionality, ontology-consistent relation types, disease-relevant tissue context, and the balance of supporting versus conflicting evidence.

## 3 Knowledge graphs as foundations for evidence chains

**FIGURE 1:**
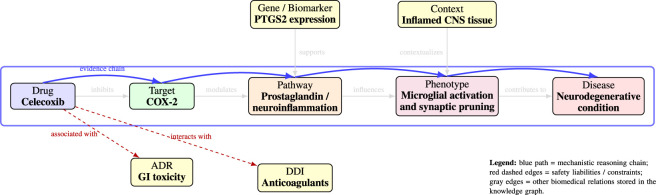
Illustrative biomedical knowledge graph for explainability-centered drug repurposing. The highlighted path shows how a candidate drug–disease association can be justified through a mechanistic evidence chain linking drug, target, pathway, phenotype, and disease, while additional nodes encode safety-relevant information such as adverse drug reactions and drug–drug interactions. This representation exemplifies how knowledge graphs support reasoning that is more transparent and pharmacologically interpretable than a prediction score alone.

Biomedical knowledge graphs provide a natural substrate for mechanistic reasoning. By organizing entities such as drugs, targets, genes, pathways, diseases, phenotypes, adverse events, and clinical observations into a semantically governed network, they enable multi-layered inference and transparent provenance tracking. Figure 1 illustrates how reasoning over an explanatory knowledge graph can be conceptualized. In the life sciences, Hetionet established one of the first comprehensive biomedical KGs designed for drug repurposing, constructed from 29 public databases and providing over 47,000 nodes and 2.25 million relationships (Himmelstein et al., 2017). Using Hetionet, Himmelstein et al., 2017 demonstrated that path-based features in such a heterogeneous network could prioritize drug–disease pairs while simultaneously producing interpretable metapaths that aligned with known biology.

Subsequent KGs expanded scale and semantic depth. SPOKE integrates data from more than 40 sources, spanning molecular, physiological, and clinical domains, resulting in over 27 million nodes and 53 million edges governed by 11 ontologies (Morris et al., 2023). Its molecular-to-clinical coverage facilitates translational inference at a scale unattainable by manual curation.

PharMeBINet offers further expansion by integrating extensive adverse drug reaction (ADR), drug–drug interaction (DDI), and genomic variant information into a multi-million-node Neo4j graph, making it particularly valuable for safety-aware reasoning in repurposing workflows (Königs et al., 2022).

Beyond their structural and semantic properties, biomedical networks have already demonstrated translational impact. During the COVID-19 pandemic, network proximity analysis identified 16 repurposable candidates and three drug combinations for SARS-CoV-2, several of which were subsequently supported by experimental or clinical evidence (Zhou et al., 2020). Notably, that approach relied on topological proximity between drug targets and disease-associated proteins rather than on explicit mechanistic paths. This distinction underscores the opportunity that path-based and evidence-chain approaches offer: not only identifying that a drug’s targets are close to a disease module, but articulating the mechanistic rationale and safety context underlying the association through multi-hop biological relationships that can be interpreted as mechanistic hypotheses.

Recent path-based repurposing methods already demonstrate that such mechanistic explanations can be operationalized in practice: explanatory rationales can be expressed as biologically interpretable paths linking compounds to diseases through intermediate genes, pathways, phenotypes, and adverse effects (Jiménez et al., 2024; Nunes et al., 2025). We therefore do not claim novelty for the mere idea of path-based explanation. Rather, our argument is that such explanations should become a primary evaluation target and should be judged not only by path plausibility, but also by uncertainty, robustness, provenance, and usefulness for downstream validation. The significance is broader than any single method: these approaches demonstrate that explainability in repurposing need not be limited to generic feature attribution, but can instead be operationalized as structured evidence chains that domain experts can inspect, challenge, and experimentally prioritize.

## 4 From neural prediction to pharmacological reasoning

Knowledge graphs provide structured biomedical context, whereas neural models provide the capacity to learn complex relational patterns from incomplete and heterogeneous data. Graph neural networks (GNNs) are therefore attractive for drug repurposing (Dai et al., 2024), but their internal reasoning remains difficult to interpret in pharmacologically meaningful terms. Model-aligned explainers such as GNNExplainer (Ying et al., 2019) and PGExplainer (Luo et al., 2020) identify prediction-relevant subgraphs, while SubgraphX (Yuan et al., 2021) explains GNN outputs by explicitly searching for important substructures using Monte Carlo tree search and Shapley-value-based scoring. Yet influence alone is not equivalent to mechanism: an influential subgraph may help explain a prediction computationally while still failing basic pharmacological expectations regarding causal direction, tissue relevance, safety, or known mechanism of action.

Beyond such neural explainers, several approaches have demonstrated that relational reasoning over knowledge graphs can produce explicitly interpretable paths or rules. Rule learning systems such as AnyBURL (Meilicke et al., 2019) derive multi-hop logical Horn rules in a bottom-up manner and expose reusable reasoning patterns underlying predictions, while extensions such as SAFRAN (Ott et al., 2021) improve their applicability by identifying redundant rules and aggregating them via non-redundant probabilistic schemes. Path-oriented approaches take a complementary perspective: methods such as MINERVA (Das et al., 2018) formulate reasoning as a reinforcement learning problem over the knowledge graph, learning to traverse multi-hop paths conditioned on a query relation, while optimization-based frameworks such as PoLo (Liu et al., 2021) explicitly search for high-quality explanatory paths connecting drugs, targets, and diseases. These approaches collectively demonstrate that explainability in relational models can be operationalized as structured multi-hop reasoning rather than simple feature attribution. However, they primarily optimize for predictive relevance, path quality, or rule aggregation and therefore address only parts of pharmacologically meaningful explainability; in the terminology of this Perspective, they do not yet jointly satisfy the full MURP requirements of mechanistic coherence, uncertainty, robustness, and provenance, which are outlined in Section 5.

**FIGURE 2:**
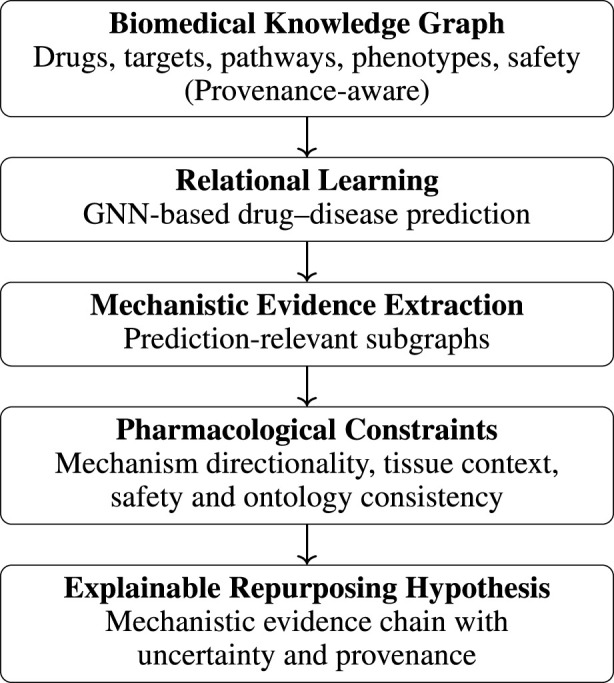
Neurosymbolic pipeline for explainability-centered drug repurposing. Structured biomedical knowledge graphs provide the biological and safety context for predictive learning. Neural models generate candidate drug–disease associations, while explanatory subgraphs articulate mechanistic rationales. Symbolic pharmacological constraints enforce biological plausibility and safety relevance, yielding evidence chains that are uncertainty-aware, provenance-rich, and suitable for experimental prioritization.

Although these methods improve transparency, they do not by themselves guarantee pharmacological coherence or mechanistic plausibility. Neurosymbolic AI could bridge that gap. Neurosymbolic AI refers to approaches that integrate neural learning from large, heterogeneous datasets with symbolic knowledge representations (such as ontologies, logical rules, or curated biological constraints) in a unified framework where symbolic knowledge actively constrains, guides, or explains model predictions (Garcez and Lamb, 2023; Bhuyan et al., 2024). In this sense, neurosymbolic systems differ from purely neural or purely rule- or path-based approaches by coupling statistical learning with explicit, interpretable reasoning constraints. Recent work such as MARS (DeLong et al., 2024) illustrates both the promise and the current limitations of this paradigm: by combining learned representations with logical rule-based reasoning over knowledge graphs, it produces more interpretable mechanism-of-action predictions, but also demonstrates that even neurosymbolic systems can remain susceptible to topological shortcuts such as degree bias if biological constraints are not carefully enforced (DeLong et al., 2025). For drug repurposing, this integration is particularly attractive. Neural explainers and rule- or path-based approaches can identify which subgraphs or reasoning chains influenced a prediction, but they do not by themselves ensure that the resulting rationale satisfies all four MURP dimensions. Figure 2 demonstrates this neurosymbolic AI pipeline.

Neurosymbolic models can address this gap by constraining or re-ranking candidate explanations according to pharmacological knowledge, such as pathway directionality, tissue-specific target expression, contraindication patterns, or ontology-consistent relations (DeLong et al., 2024; Hossain and Chen, 2025). For example, a GNN may propose a drug–disease association on the basis of multi-hop connectivity, while a symbolic layer retains only those explanatory paths that remain consistent with known mechanism-of-action and safety knowledge. In this way, neurosymbolic AI offers not only greater transparency, but also a stronger bridge between statistical prediction and pharmacological reasoning (Garcez and Lamb, 2023; Drancé, 2022).

Yet mechanistic plausibility alone is insufficient: if explanations are unstable, overconfident, or weakly supported, they cannot reliably guide experimental follow-up. This motivates the evaluation framework introduced in the next section.

## 5 Mechanistic coherence, uncertainty, robustness, and provenance as essential components

BOX 2 **Definition: Pharmacologically Meaningful Explainability**

We define *explainability* in AI-driven drug repurposing as a multi-dimensional assessment profile:

$$\text{MURP}\left(h\right)=\left\langle \;M\left(h\right),\;U\left(h\right),\;R\left(h\right),\;P\left(h\right)\;\right\rangle$$

where h denotes a candidate repurposing hypothesis and:

M (Mechanistic coherence): the presence of an explicit, biologically plausible evidence chain linking a drug to a clinical outcome (e.g., drug → target → pathway → phenotype → outcome).

U (Uncertainty): quantified epistemic and data-driven uncertainty associated with both predictions and explanatory links.

R (Robustness): stability of predictions and explanations under perturbations of model parameters, graph structure, or data provenance.

P (Provenance): traceability of each explanatory element to its underlying data sources, curation level, and evidentiary support.

Each dimension is assessed qualitatively or semi-quantitatively in a domain-appropriate manner. The tuple representation is deliberate: we do not propose collapsing these dimensions into a single scalar score, because their relative importance varies by context of use (e.g., early exploration vs. regulatory-facing decision-making). An explanation that is deficient in any single dimension should be treated with caution, regardless of predictive performance. For brevity, we refer to these four dimensions collectively as MURP.

For AI-based drug repurposing, explainability is only useful if the proposed rationale is accompanied by an assessment of how reliable, stable, and traceable that rationale actually is (Perdomo-Quinteiro and Belmonte-Hernández, 2024). A mechanistic explanation may appear biologically plausible, yet still rest on weak evidence, unstable graph structure, or overconfident predictions. In this sense, these properties are not secondary technical considerations, but essential conditions for deciding whether a computational hypothesis is sufficiently trustworthy to justify experimental follow-up.

To make this framework more operational, each component should be assessed in practice. *Mechanistic coherence* can be evaluated by checking whether an extracted chain respects ontology-consistent relation types, biological directionality, and tissue or cell-type relevance, and whether domain experts judge the chain to be pharmacologically plausible. *Uncertainty* should be reported at both the prediction level and the explanatory-edge level, so that users can distinguish broadly confident hypotheses from those dependent on fragile links. *Robustness* should be tested by graph perturbation, ablation of low-provenance edges, alternative graph snapshots, or variation across model seeds, asking whether the same core rationale persists. *Provenance* should identify the source database, evidence type, curation status, and supporting literature for each explanatory edge.

Uncertainty estimation is particularly important because high predictive scores can mask substantial epistemic or data-driven uncertainty (Guo et al., 2017). In drug repurposing, this distinction matters directly for prioritization: a candidate supported by a coherent mechanistic explanation but associated with high uncertainty should be interpreted differently from one supported by similarly plausible evidence with stronger calibration and greater stability. Methods such as deep ensembles have shown strong performance in producing better-calibrated uncertainty estimates and in detecting distributional shift (Lakshminarayanan et al., 2017), while Monte Carlo dropout offers a practical approximation of model uncertainty within standard neural architectures (Gal and Ghahramani, 2016). Rather than treating uncertainty as an abstract property of the model, repurposing workflows should expose it at the level of explanatory edges and paths, thereby allowing researchers to distinguish relatively robust evidence chains from predictions that remain mechanistically fragile.

Robustness is equally important because even an apparently interpretable explanation may depend on unstable graph topology, low-quality relations, or isolated shortcuts learned by the model. This is especially relevant in biomedical KGs, where the completeness and quality of edges vary substantially across domains, diseases, and data sources. Robustness analysis should therefore test whether an explanation remains stable when low-provenance relations are removed, when subgraphs are perturbed, or when alternative KG snapshots are used (Ying et al., 2019; Luo et al., 2020; Agarwal et al., 2023). From a pharmacological perspective, this matters because a rationale that collapses under minor perturbation is unlikely to provide a reliable basis for mechanistic interpretation or experimental design.

Provenance adds a further layer of credibility by clarifying where each element of an explanation originates and how much evidentiary weight it should carry. In biomedical KGs, explanatory paths may combine manually curated relations, ontology-grounded mappings, inferred links, and literature-derived associations, all of which differ in reliability and interpretability (Königs et al., 2022; Himmelstein et al., 2017). Provenance tracking is therefore not only a matter of technical reproducibility, but also of scientific judgment: it allows researchers to distinguish well-supported mechanistic links from weaker associations that require additional scrutiny. Conflicting biological evidence should likewise be made explicit rather than hidden. When supporting and contradicting chains coexist, the prediction should be flagged for expert adjudication instead of being presented as a singular mechanistic truth.

Taken together, these four dimensions determine whether an explanation is merely computationally plausible or credible enough to support pharmacological follow-up and should accordingly be treated as integral to model evaluation rather than as optional post-hoc diagnostics.

## 6 Toward a pharmacology-centered standard for AI repurposing

If explainability is to function as a criterion of pharmacological credibility rather than a post-hoc reporting accessory, the field needs a clearer standard for model development, evaluation, and communication. We argue that AI-based repurposing systems should be designed to produce, alongside each predicted drug–disease association, a MURP-compliant evidence chain: a mechanistically interpretable path annotated with uncertainty, robustness, and provenance information. Under such a standard, explainability is not a supplementary visualization layer but part of what constitutes a usable repurposing hypothesis.

The starting point is a carefully selected and versioned KG substrate, chosen for its coverage of drugs, targets, pathways, phenotypes, clinical signals, and safety-relevant relations. Heterogeneous GNNs or hybrid neurosymbolic models then learn relational patterns for link prediction while being instrumented for explanation extraction. Explanatory subgraphs are produced via GNNExplainer or PGExplainer and subsequently refined through symbolic constraints and uncertainty estimation. The final product for each predicted drug–disease pair is a *structured evidence chain*: a serialized, human-interpretable subgraph that highlights mechanistic steps, indicates provenance of each edge, communicates uncertainty, and incorporates safety considerations such as adverse drug reactions, drug–drug interactions, and contraindications – a capability especially well supported by ADR-enriched KGs such as PharMeBINet (Königs et al., 2022).

Evaluation should therefore combine conventional predictive metrics with the MURP dimensions introduced above, plus explicit assessment of safety relevance and experimental actionability. A model that performs well statistically but fails these tests may remain computationally impressive while being weak as a pharmacological decision-support system.

The importance of these criteria also varies by context of use. In early exploratory prioritization, a statistical signal may still be useful for hypothesis generation even before a detailed mechanism is available. Mechanistic explainability becomes more important when predictions are used to prioritize costly experiments, assess safety risks, support translational decisions, or inform regulatory-facing deliberation. In other words, the stronger the downstream decision consequences, the stronger the requirement for structured, uncertainty-aware, provenance-rich explanation.

This integrated approach not only enables rigorous scientific assessment but also supports a more collaborative, human-in-the-loop research process. Pharmacologists and domain experts can review, prune, or augment evidence chains; their feedback can then be incorporated as constraints, ranking signals, or adjudication criteria, helping align model outputs with the forms of reasoning that experimental pharmacology actually requires. In practical terms, such a framework would allow repurposing predictions to be prioritized not only by score, but by the quality of their mechanistic rationale, safety context, evidentiary robustness, and readiness for downstream validation.

## 7 Illustrative vignettes

To clarify how the proposed framework relates to practice, we distinguish between literature-based examples and hypothetical scenarios.

A useful counterexample is the discovery of halicin by deep learning (Stokes et al., 2020). In that case, a model identified a structurally novel antibiotic candidate with limited mechanistic explainability at the time of prediction, yet the finding gained strong credibility through extensive experimental validation, including activity against multiple pathogens and efficacy in murine infection models. This case is important because it shows that mechanistic explainability is not the only route to a valuable discovery in early-stage exploration. At the same time, subsequent work has shown that explainable deep learning can help identify structural rationales underlying antibacterial activity, suggesting that explainability and experimental validation are complementary rather than competing goals (Wong et al., 2024).

A second literature-based example is provided by recent network-medicine work in Alzheimer’s disease (Bykova et al., 2026). There, network-based prediction identified doxycycline and irbesartan as repurposing candidates, and large-scale real-world patient data were then used to assess whether prescription exposure was associated with reduced disease incidence. This paradigm is instructive because it combines computational prioritization with translational corroboration rather than treating either one as sufficient on its own. It therefore exemplifies the broader argument advanced here: pharmacological credibility is strongest when mechanistic reasoning, data integration, and empirical follow-up reinforce one another.

The following three scenarios are illustrative rather than empirical findings, and they are intended only to show how explainability-centered workflows can alter decision-making.

In the first hypothetical scenario, a black-box GNN ranks a COX-2 inhibitor as a promising candidate for a neurodegenerative condition. Lacking mechanistic visibility, the suggestion appears arbitrary. An explainable KG-based model, by contrast, reveals a multi-hop chain linking COX-2 inhibition to microglial PGE_2_ modulation, synaptic pruning pathways, and neuroinflammation signals observed in clinical cohorts. It also surfaces known gastrointestinal adverse drug reactions and a potential drug–drug interaction with anticoagulants. This enriched rationale informs dose selection, safety monitoring strategies, and downstream experimental planning, which would be inaccessible from a probability score alone.

In the second hypothetical scenario, a neurosymbolic model proposes a JAK/STAT-modulating compound for fibrotic disease. The explanation identifies a critical protein–protein interaction that connects the compound’s primary target to TGF- β signaling. However, uncertainty estimation highlights this edge as low-confidence due to limited provenance. Researchers may then prioritize validating this molecular interaction before committing resources to *in vivo* studies. Again, mechanistic transparency and uncertainty together guide more efficient and disciplined experimentation.

In the third hypothetical scenario, a GNN achieves high AUROC for a predicted drug–disease association driven by dense network connectivity. Inspection of the extracted evidence chain reveals a missing mechanistic link: the proposed target is not expressed in the disease-relevant tissue, and the inferred pathway direction contradicts established disease biology. In addition, safety annotations surface a known adverse effect that directly exacerbates a core clinical feature of the disease. Despite strong benchmark performance, the prediction is rejected as pharmacologically non-viable due to the absence of a coherent mechanism and unresolved safety conflicts.

## 8 Discussion

The central message of this Perspective is not that benchmark metrics are irrelevant, nor that every useful discovery must begin with a complete mechanistic account. Rather, it is that pharmacological credibility cannot be inferred from predictive performance alone. Drug repurposing decisions require more than ranked predictions: they require hypotheses that can be interrogated in terms of mechanism of action, pathway relevance, tissue context, safety liabilities, and translational plausibility. From this perspective, the limitation of many current AI approaches is not simply opacity in the abstract, but the absence of explanations that are usable within pharmacological reasoning and experimental decision-making.

This point should be calibrated across stages of the translational pipeline. In early exploratory discovery, a statistically strong signal may still be valuable as a hypothesis-generating starting point even before a detailed mechanistic rationale is available. The halicin case underscores this possibility (Stokes et al., 2020). By contrast, when predictions are used to prioritize costly experiments, justify biological plausibility, assess safety risks, support translational investment, or inform regulatory-facing use, mechanistic explainability becomes substantially more important. In these contexts, opaque rankings provide too little guidance about why a candidate might work, how it might fail, and which liabilities should be monitored.

Network medicine approaches (Barabási et al., 2011) illustrate this complementarity: network proximity–based screening identified sildenafil as a candidate for Alzheimer’s disease, later supported by real-world patient data and iPSC experiments (Fang et al., 2021), and multiple repurposable drugs for SARS-CoV-2 under pandemic time pressure (Zhou et al., 2020). In both cases, translational credibility emerged not from computational prediction alone, but from the reinforcement of mechanistic rationale by empirical follow-up.

The value of a computational rationale is not only that it is interpretable after the fact, but that it helps determine which assays to run, which liabilities to monitor, which mechanistic links to validate first, and which predictions are too weakly supported to justify immediate follow-up. In this sense, explainability is inseparable from resource allocation in translational pharmacology: it helps convert broad computational possibility into disciplined experimental prioritization. But the ultimate test of pharmacological credibility remains empirical, whether through *in vitro* target engagement, phenotypic assays, *in vivo* studies, clinical observation, or real-world evidence.

Path-based and KG-based explainability for drug repurposing already exists, and important reviews have highlighted the opportunities and limitations of explainable AI (XAI) in drug discovery more broadly (Jiménez et al., 2024; Nunes et al., 2025; Lavecchia, 2025; Tanoli et al., 2025). Our intended contribution is therefore not to claim invention of evidence-chain reasoning itself, but to argue that pharmacology-aligned explanation – enriched by the above-mentioned dimensions – should become a central evaluation target for AI-enabled repurposing systems, especially when they are used for translationally consequential decisions.

This Perspective has several limitations. First, it does not present a new benchmark, executable pipeline, or prospective experimental study; its purpose is conceptual and normative rather than empirical. Second, the literature coverage is representative rather than systematic. Third, the proposed framework will require adaptation across disease areas, data modalities, and contexts of use. These limitations also define an agenda for future work: prospective studies should test whether evidence-chain-based evaluation improves experimental hit rates, resource allocation, safety assessment, or translational decision quality compared with accuracy-first baselines.

## 9 Conclusion

AI-enabled drug repurposing should not equate high predictive performance with pharmacological credibility. Predictions become more useful for translation when accompanied by inspectable rationales linking drugs to outcomes through targets, pathways, phenotypes, safety context, uncertainty, robustness, and provenance. Knowledge graphs, graph-based learning, and neurosymbolic approaches provide a practical foundation for such evidence-chain reasoning. Their value, however, depends on evaluation standards that reward not only benchmark accuracy, but also mechanistic coherence and experimental usefulness. Prediction without rationale may support exploration, but it is often insufficient for translation. Explainability without uncertainty invites overconfidence. Progress should therefore be measured by how effectively models generate hypotheses that scientists can interrogate, experiments can validate, and decision-makers can trust.

## Data Availability

The original contributions presented in the study are included in the article/supplementary material, further inquiries can be directed to the corresponding author.

## Footnotes

1
https://www.fda.gov/about-fda/artificial-intelligence-drug-development/guiding-principles-good-ai-practice-drug-development
